# Characterizing Social Media Messages Related to Underage JUUL E-Cigarette Buying and Selling: Cross-Sectional Analysis of Reddit Subreddits

**DOI:** 10.2196/16962

**Published:** 2020-07-20

**Authors:** Hejing Liu, Qiudan Li, Yongcheng Zhan, Zhu Zhang, Daniel D Zeng, Scott J Leischow

**Affiliations:** 1 The State Key Laboratory of Management and Control for Complex Systems Institute of Automation Chinese Academy of Sciences Beijing China; 2 School of Artificial Intelligence University of Chinese Academy of Sciences Beijing China; 3 Shenzhen Artificial Intelligence and Data Science Institute (Longhua) Shenzhen China; 4 Orfalea College of Business California Polytechnic State University San Luis Obispo, CA United States; 5 College of Health Solutions Arizona State University Phoenix, AZ United States

**Keywords:** JUUL, e-cigarette, Reddit, cross-sectional analysis, electronic nicotine delivery system, underage JUUL use

## Abstract

**Background:**

Stopping the epidemic of e-cigarette use among youth has become the common goal of both regulatory authorities and health departments. JUUL is currently the most popular e-cigarette brand on the market. Young people usually obtain and exchange information about JUUL with the help of social media platforms. Along with the rising prevalence of JUUL, posts about underage JUUL buying and selling have appeared on social media platforms such as Reddit, which sharply increase the risk of minors being exposed to JUUL.

**Objective:**

This study aims to analyze Reddit messages about JUUL buying and selling among the users of the UnderageJuul subreddit, and to further summarize the characteristics of those messages. The findings and insights can contribute to a better understanding of the patterns of underage JUUL use, and help public health officials provide timely education and guidance to minors who have intentions of accessing JUUL.

**Methods:**

We used a novel cross-subreddit method to analyze the Reddit messages on 2 subreddits. From July 9, 2017, to January 7, 2018, we collected data from the UnderageJuul subreddit, which was created for underage JUUL use discussion. The data set included 716 threads, 2935 comments, and 844 Reddit users (ie, Redditors). We collected our second data set, comprising 23,840 threads and 162,106 comments posted between July 9, 2017, and January 8, 2019, from the JUUL subreddit. We conducted analyses including the following: (1) annotation of users with buying/selling intention, (2) posting patterns discovery and topic comparison, and (3) posting activeness observation of discovered Redditors. Term frequency–inverse document frequency and regular expression-enhanced keyword search methods were applied during the content analysis to extract the posting patterns. The public posting records of the discovered users on the JUUL subreddit during the year after the UnderageJuul subreddit was shut down were analyzed to determine whether they were still active and interested in obtaining JUUL.

**Results:**

Our study revealed the following: (1) Among the 716 threads on the UnderageJuul subreddit, there were 214 threads related to JUUL sale and 168 threads related to JUUL purchase, which accounted for 53.5% (382/714) of threads. (2) Among the 844 Redditors of the UnderageJuul subreddit, 23.82% (201/844) of users were annotated with buying intention, and 21.10% (178/844) of users were annotated with selling intention. There were 34 users with buying/selling intention that self-reported as being <21 years old. (3) The most common key phrases used in selling threads were “WTS,” “want to sell,” “for sale,” and “selling” (154/214, 72.0%). The most common key phrases used in buying threads were “look for/get JUUL/pods” (58/168, 34.5%) and “WTB” (53/168, 31.5%). (4) The most important concern that UnderageJuul Redditors had in obtaining JUULs was the price (311/1306, 23.81%), followed by the delivery service (68/1306, 5.21%). (5) The most popular flavors among the users with buying/selling intention were mango, cucumber, and mint. The flavor preferences remained consistent on both subreddits. Adverse symptoms related to the mango flavor were reported by 3 users on the JUUL subreddit. (6) In total, 24.4% (49/201) of users wanted to buy JUULs and 46.6% (83/178) of users wanted to sell JUULs, including 11 self-reported underage users, who also participated in the discussions on the JUUL subreddit. (7) Within one year of the UnderageJuul subreddit shutting down, there were 40 users who continued to post 186 threads on the JUUL subreddit, including 10 threads indicating buying/selling willingness that were posted shortly after the UnderageJuul subreddit was closed.

**Conclusions:**

There were overlapping users active in the JUUL and UnderageJuul subreddits. The buying/selling-related content appeared in multiple venues with certain posting patterns from July 9, 2017, to January 7, 2018. Such content might lead to a high risk of health problems for minors, such as nicotine addiction. Based on these findings, this study provided some insights and suggestions that might contribute to the decision-making processes of regulators and public health officials.

## Introduction

The use of Electronic Nicotine Delivery Systems (ENDS; also called “e-cigarettes”) has been increasing rapidly among youth. In 2019, a cross-sectional survey with 19,018 participants showed the prevalence of self-reported current e-cigarette use was 27.5% among US high school students and 10.5% among middle school students [1]. At present, JUUL (by JUUL Labs) is the most popular brand, with more than 70% of the market share of e-cigarettes [2]. The appealing flavors and its relaxing effect cause potential intention and actual use among nonsmoking teenagers [3-5]. In addition, because of the lax regulatory environment in the United States, unregulated constituents in e-liquids as well as high nicotine delivery have led to concerns about addiction potential and pulmonary risks from the use of ENDS products [6-8]. To protect the physical health of young people, in April 2018, the Food and Drug Administration (FDA) issued a statement on preventing teenagers from using and buying JUUL and similar products [9]. As the FDA indicated [9], JUUL was more difficult for parents and teachers to recognize and detect than other e-cigarette products. Therefore, increased efforts to strengthen the regulation of minors' access to JUUL have been implemented.

JUUL’s marketing heavily relied on social media platforms like Twitter and Instagram [10]. As a result, young people read and spread information about JUUL with the help of social media platforms [11]. Common JUUL discussions on social media included flavor preference [12-14], use experience [12-16], use location [12,14], purchasing methods [17], etc. Allem et al [12] found that JUUL use occurred in school locations, such as classrooms, bathrooms, libraries, and gyms. Brett et al [14] found teenagers used JUUL because the use of this product was part of a popular trend and to feel the “buzz” (ie, a feeling caused by nicotine). Multiple researchers have noted that adolescents prefer the mango and mint flavors [14,17,18]. Li et al [19] found that the fruit flavor was associated with multiple adverse symptoms, including cough, headache, and lung tightness, though the causal relationship is unclear. With the rise of youth exposure to JUUL, Zhan et al [17] analyzed their purchasing methods. They found that peer-to-peer purchase and shipping was the most common method for UnderageJuul subreddit users.

As an online forum that allows users to create communities, Reddit has multiple sections (also called “subreddits”) related to JUUL discussion. Previous research determined that underage Redditors post about their JUUL use on Reddit [20]. To better understand and surveil youth JUUL use, researchers have conducted analyses on Reddit data focusing on underage JUUL users and their posting content, such as topic discovery [14,15], age and location distribution [17], popular flavor discovery [15,17], and negative symptoms analysis [19].

Previously, there was a public subreddit on Reddit called UnderageJuul, which was dedicated to discussing underage JUUL use. This subreddit was created in July 2017 and had almost 1000 users at its peak. Right before it was shut down in January 2018, the UnderageJuul subreddit was gaining 8 new members per day [20,21]. Previous studies conducted qualitative analysis on the UnderageJuul subreddit and found that posts in this subreddit included content such as self-reported underage JUUL users seeking sellers, sale advertisements without age verification, and adult users buying for minors to earn money, researchers thought such trade-related content indicated that minors had increased access to JUUL, and there might be other similar communities on social media platforms [17,20,21]. However, the characteristics of the buying/selling-related messages among the users of the UnderageJuul subreddit have not been further identified.

This study aimed to analyze the content on the UnderageJuul and JUUL subreddits that indicated JUUL buying and selling, which provides a more comprehensive understanding of underage JUUL use. Specifically, this study was designed to address the following questions:

First, what are the posting patterns of messages related to JUUL buying and selling on the UnderageJuul subreddit? The “posting patterns” refer to the common key phrases, way of expression, variants, or abbreviations the Redditors used when publishing buying/selling-related posts. For instance, Redditors usually use “look for/need + juul/pods” or “WTB” (short for “want to buy”) to express their buying willingness. Discovering users’ posting patterns could enable researchers to extract the core features of this kind of message, and help regulators continue to identify similar content on social media, thus contributing to the prevention of minors’ access to JUULs.

Second, what are the concerns of the users of the UnderageJuul subreddit when they want to buy or sell JUULs? While UnderageJuul users had a willingness to buy and use JUULs, they also expressed concerns regarding JUULs, such as the choice of flavors. Given this subreddit was created to discuss underage JUUL use, identifying the concerns of its users has the potential to uncover specific characteristics of minors or others using that subreddit who intend to buy or sell JUUL. Understanding the topics and concerns discussed in this subreddit could be useful for health departments or educators responsible for the development and implementation of education programs on potential ENDS risk, as well as for regulatory agencies responsible for developing media and educational programs for young people.

Third, will users of the UnderageJuul subreddit cross-publish their comments, questions, and concerns on multiple subreddits at the same time? Will they continue to be active after the UnderageJuul subreddit is closed? We adopted the evaluation of “posting activeness” to address these questions, which involves counting the total number of different posts by users [22]. We chose the JUUL subreddit (ie, the general JUUL discussion subreddit) as a contrast. The posting activeness across subreddits will be compared during two time periods: the time period when UnderageJuul was accessible, and the year after UnderageJuul was shut down. This analysis could provide a deeper understanding of the buying and selling behavior of UnderageJuul users, including the extent to which shutting down one social media channel impacts information-sharing on other platforms, which could have significance for both understanding the breadth and scope of social media networks and the implementation of social media–based educational programs.

By answering the 3 questions above, we hope to provide valuable information to regulators to help them establish practices and policies to minimize adolescent e-cigarette use and safeguard their health. Note that the user analysis in this study was for the users who had public posting records on the UnderageJuul subreddit. The ages of underage users were based on their self-reported age information.

## Methods

### Overview

**Figure 1:**
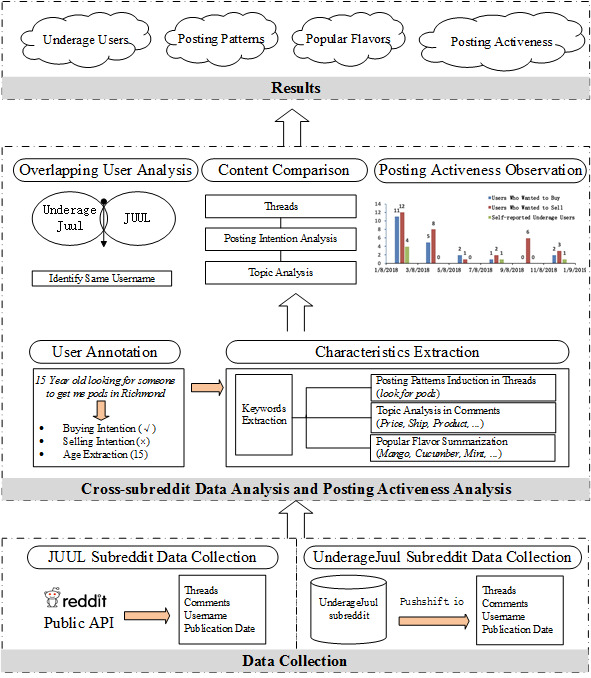
Framework for cross-subreddit analyses on Reddit. API: application programming interface.

We applied a novel cross-subreddit method to analyze the content related to JUUL buying and selling, with a focus on comparing those using the UnderageJuul subreddit (which was originally designed for young people to discuss the use of JUULs, but soon became a platform to explore and share ways to illegally obtain JUULs) with those using the general JUUL subreddit (which is the main Reddit channel for discussing general JUUL-related topics). Figure 1 shows the framework for our cross-subreddit analyses. It consists of three components: data collection; cross-subreddit content and posting activeness analysis; and results.

### Data Collection

The data used in this research consisted of two parts: data from the UnderageJuul subreddit and data from the JUUL subreddit.

#### UnderageJuul Subreddit Data Collection

Since the UnderageJuul subreddit was shut down by the time we began our analysis, we collected data from the UnderageJuul subreddit through the application programming interface (API) provided by pushshift.io [23], which is a website that stores all publicly available Reddit threads and comments. The UnderageJuul subreddit was created on July 9, 2017, and was shut down on January 8, 2018. We obtained the complete UnderageJuul subreddit posting and user information data set from that period, including 716 threads, 2935 comments, and 844 Redditors.

#### JUUL Subreddit Data Collection

We collected data from the JUUL subreddit via Reddit’s API [24] from July 9, 2017, to January 8, 2019, which covered two continuous time periods: (1) the same time period of the UnderageJuul data set (from July 9, 2017, to January 7, 2018), and (2) the year after the end of UnderageJuul activities (from January 8, 2018, to January 8, 2019). The first data set (ie, the JUUL-overlap data set) contained 3978 threads, 31,589 comments, and 5785 Redditors. The second data set (ie, the JUUL-afterwards data set) contained 19,862 threads, 130,517 comments, and 18,460 Redditors. Table 1 shows a detailed description of these three data sets.

Note that the data we used in this research were all publicly available on Reddit, and no personal information (eg, account profile) was included. The usernames contained in these data sets are not presented in this paper, for the privacy protection of the Redditors involved in this study.

**Table 1:** Data set description.

Data set	Time span	Threads, n	Comments, n	Redditors, n	Threads per Redditor, n	Comments per Redditor, n
UnderageJuul	07/09/2017-01/07/2018	716	2935	844	1.95	4.11
JUUL-overlap	07/09/2017-01/07/2018	3978	31,589	5785	2.45	5.95
JUUL-afterwards	01/08/2018-01/08/2019	19,862	130,517	21,186	2.49	7.07

### Data Analysis

#### Overview

The data analyses consisted of three steps. The first step was to annotate the users on the UnderageJuul subreddit that indicated buying/selling intention. Second, the text features were extracted to discover the posting patterns in their posting content. Finally, the activeness of the Reddit accounts was analyzed. We used a cross-subreddit analysis method in this study, thus the results of each step were all compared with the content on the JUUL subreddit.

#### User Analysis Across Subreddits

##### Overview

We first set the rules for annotation, then manually labeled Redditors who wanted to buy or sell JUUL on the UnderageJuul subreddit. Based on the results of user annotation on the UnderageJuul subreddit, the overlapping users were detected to discover how many of these users also participated in the JUUL subreddit during the same time period.

##### Step 1: User Annotation on the UnderageJuul Subreddit

First, two annotators labeled the users who wanted to buy or sell JUULs according to the descriptions in Table 2. Users interested in buying or selling usually post a specialized thread to attract sellers or to advertise the product to be sold, so we went through all threads on UnderageJuul subreddit. For example, when a Redditor posted a thread saying “[WTB] Looking to buy blue Juul and cucumber pods for under $75,” he/she was labeled as a user who wanted to buy JUULs; when a Redditor posted a thread saying “I am selling Juul warranty codes, …, 25$ per code, 20$ per code (if you buy 3 or more at once),” he/she was labeled as a user who wanted to sell JUULs. The threads were also tagged with corresponding labels. A Redditor could be annotated as a user who wanted to buy JUULs as well as a user who wanted to sell JUULs, if he/she posted multiple threads indicating both buying and selling intentions. Then, we annotated all comments below these labeled threads to discover users who did not post themselves but replied to those threads and showed buying/selling willingness. The Cohen κ coefficient of the annotation from two annotators was 0.83. We next extracted age information from the posts of these users to discover underage JUUL users. Since the US federal minimum age for sale of tobacco products had been raised from 18 to 21 years (ie, the Tobacco 21 legislation [25]), we labeled Redditors who self-reported that they were under 21 years old as the self-reported underage users. We used regular expressions to extract phrases like “# years old” or “I’m #” to detect age information of corresponding users. Some users did not mention a specific age but had expressions like “I’m underage” in their posts. These users were also labeled as self-reported underage users.

**Table 2:** Annotation categories and description.

Label	Description
User who wanted to buy JUULs	User who wanted to purchase JUULs peer-to-peer or face-to-face, asked others to buy JUULs for him/her, or wanted to buy a JUUL replacement code to get JUULs.
User who wanted to sell JUULs	User who wanted to sell JUULs to others through shipping or face-to-face trade, buy JUUL for others for a fee, or sell JUUL replacement codes.
Self-reported underage user	User who wanted to buy or sell JUULs and had a self-report age under 21 years old or a description indicating underage.

##### Step 2: Overlapping User Analysis on the JUUL Subreddit

The username is the unique identifier for each Redditor [26]. We compared the public posting records of these users on the two subreddits UnderageJuul and JUUL (ie, the JUUL-overlap data set). Redditors with the same usernames were regarded as overlapping users.

#### Content Analyses and Comparison

##### Overview

The following content analyses consisted of two steps. First, we extracted the common key phrases and words from the user-generated content to summarize the regular posting patterns. Second, we compared the overlapping users’ posts between the UnderageJuul subreddit and the JUUL subreddit.

##### Step 1: Posting Patterns Extraction on the UnderageJuul Subreddit

First, the textual features needed to be extracted from users’ posts. To achieve this, we used term frequency–inverse document frequency to uncover important words in each post automatically. Based on the frequency of these important words, we adopted regular expression-enhanced keyword search [27] on each post to discover key phrases. The trigrams near the important words were extracted from the buying/selling-related threads. The key phrases were determined according to the frequency rank and whether the expression was complete. For example, the key phrase “need juuls/pods” was determined because it was a complete expression and had a top rank in the search results of the word “need.” We manually classified the important words and key phrases into several posting patterns based on semantics and phrase structure.

##### Step 2: Content Comparison on the JUUL Subreddit

We compared the user-generated contents of overlapping users between the JUUL and UnderageJuul subreddits to discover whether these users also published trade-related content on the JUUL subreddit, and whether their concerns remained consistent. For the first perspective, each thread posted by the overlapping users on the JUUL subreddit was automatically labeled if it implied a willingness to buy or sell JUULs using the posting patterns discovered in Step 1. For the second perspective, we conducted a qualitative content analysis on users’ posts on both subreddits. To acquire more detailed and specific concerns, our analysis was applied to all the comments under trade-related threads on the UnderageJuul subreddit, as well as all the content they posted on the JUUL subreddit (ie, threads and comments). We used the inductive category development method [28] to generate the topics based on the posts’ semantics and JUUL background information. For example, expressions like “v1” and “v2” represented versions of JUUL device. When they were identified in the posts, they were categorized with other words (eg, “leak,” “refill”) that were previously found to be related to JUUL devices, regarded as the “device description” topic. Because flavor is one of the most important factors in ENDS products that appeal to teenagers, we specifically counted the occurrences of JUUL’s seven flavors (ie, mango, mint, cucumber, menthol, fruit, crème, and tobacco) among the discussions of these users to discover their preference.

#### Posting Activeness Observation

Finally, the online posting activeness of these users on the JUUL subreddit was observed during the year after the UnderageJuul subreddit was shut down. We analyzed the posting activeness of users to find whether they continued to express a willingness to buy or sell in other communities after the UnderageJuul subreddit was closed. Specifically, we searched through the JUUL-afterwards data set to count the number of threads and comments posted by these users, recorded their posting dates to get the posting trend changes, and analyzed their posts. Thus, we manually checked their content to determine whether their posts showed the intention to buy/sell JUUL.

## Results

### User Analyses Across Subreddits

Among 844 Redditors on the UnderageJuul subreddit, we discovered 201 users who wanted to buy JUULs, 178 users who wanted to sell JUULs, and 45 users with both intentions. Among the 334 discovered users, there were 34 users that were self-reported underage. Of these, 30 indicated a willingness to purchase and 4 were willing to sell JUUL. Table 3 shows the proportion of each self-reported age group. The “teenager, age unknown” group represents users that did not mention a specific age but used expressions like “I’m underage” in their posts.

**Table 3:** Groups of discovered users with self-reported age.

User age (years)	Number of discovered users
≤15	5
16-18	14
19-21	3
Teenager, age unknown	5

After analyzing the users across subreddits, we found 49 of 201 (24.4%) users who wanted to buy JUULs and 83 of 178 (46.6%) users who wanted to sell JUULs also participated in discussions on the JUUL subreddit. Among the overlapping users, 11 underage users (8 users with buying willingness and 3 users with selling willingness) were discovered. Table 4 shows the detailed statistics of the overlapping users.

We also counted the threads and comments posted by the discovered users on each subreddit. They posted 382 threads in the UnderageJuul subreddit and there were 1306 comments under their threads. In addition, they also posted 123 threads and 1600 comments in the JUUL subreddit. Table 5 shows the detailed statistics of their posts. We ranked the activeness of all overlapping users according to their posting amount. The most active self-reported underage user published 16 threads and 96 comments on the JUUL subreddit, as well as 1 thread and 44 comments on the UnderageJuul subreddit. There were 128 Redditors that posted 182 replies to this user.

**Table 4:** Statistics of the discovered users and overlapping users.

User types	UnderageJuul subreddit, n/N (%)	JUUL-overlap subreddit, n/N (%)
Users who wanted to buy JUULs	201/844 (23.8)	49/201 (24.4)
Users who wanted to sell JUULs	178/844 (21.1)	83/178 (46.6)
Self-reported underage users	34/334 (10.2)	11/34 (32.4)

**Table 5:** Statistics of posts from discovered users and overlapping users.

Data set	Users who wanted to buy JUULs			Users who wanted to sell JUULs		
	Threads, n/N	Comments, n/N	Total, n	Threads, n/N	Comments, n/N	Total, n
UnderageJuul	168/716	180/1306	348	214/716	238/1306	452
JUUL-overlap	39/3978	447/31,589	486	84/31,589	1153/31,589	1237

### Content Analyses and Comparison

#### Posting Patterns Analysis

We summarized 10 patterns observed in the threads posted by the users discovered on the UnderageJuul subreddit. Table 6 presents each pattern and its ratios. Since one thread may contain multiple key phrases, the total post numbers of the key phrases groups are greater than the total number of posts mentioned in Table 5.

The posting patterns in the threads from users who wanted to buy JUULs all expressed buying willingness directly. They usually included “looking for juul/pods” (58/168, 34.5%) and “want to buy (WTB)” (53/168, 31.5%) in the title, then described the buying requests in the body, such as the flavor and the location. An example is “Need Pods In Orlando Area. Just an underaged kid looking for pods in the Orlando area any flavor.” The body of these threads also contained the key phrases like “need juul/pods” or “need help for getting juul/pods” (36/168, 21.4%). Alternative expressions were also used to show buying willingness, such as “anyone selling juul/pods” and “anyone know seller in somewhere” (28/168, 16.7%).

**Table 6:** Regular posting patterns of discovered users on the UnderageJuul subreddit.

User type, topic number, and key phrases			Post, n (%)
**Users who wanted to buy JUULs (n=168)**			
	1	WTB^a^, want to buy, buy juul/pods	53 (31.5)
	2	Need juul/pods, need help, need seller	36 (21.4)
	3	Look for juul/pods, get juul/pods	58 (34.5)
	4	Anyone selling, anyone know seller	28 (16.7)
	5	Other	57 (34.0)
**Users who wanted to sell JUULs (n=214)**			
	1	WTS^b^, want to sell, for sale, selling	154 (72.0)
	2	Ship, discreet	97 (45.3)
	3	Charger, refill, starter	55 (25.7)
	4	Sealed^c^, unboxing, unopen, brand new	40 (18.7)
	5	Other	39 (18.2)

^a^WTB: want to buy.

^b^WTS: want to sell.

^c^“Sealed” indicates that the product is new and has not been opened.

The posting patterns in the threads from users who wanted to sell JUULs included multiple kinds of information. They usually contained key phrases such as “WTS,” and “something for sale” (Pattern 1, 154/214, 72.0%) in the title to draw attention. Pattern 2 (97/214, 45.3%) was about shipping services, such as the shipping scope and discretion. Pattern 3 (55/214, 25.7%) involved a description of the product, including the accessories. If the product was brand-new, that was emphasized (40/214, 18.7%). For instance, a user who wanted to sell JUULs posted the following thread: “[WTS] JUUL V3 Starter Kit $40 in BTC. Brand new, sealed starter kit. Will ship quickly and discreetly.”

Based on the above posting patterns, we further analyzed the threads posted by the overlapping users on the JUUL subreddit and found 17 threads with buying purpose and 18 threads with selling purpose.

#### Content Comparison

We summarized the 15 topics discussed by the discovered users in the UnderageJuul and JUUL subreddits. Table 7 shows the 10 topics discussed in the UnderageJuul subreddit and the 5 topics discussed in the JUUL subreddit.

**Table 7:** Topic comparison of the UnderageJuul and JUUL subreddits.

Subreddit, topic number, and topic			Key phrases	Posts, n (%)
**UnderageJuul** **subreddit (n=1306)**				
	1	Price	Price, how much, $number	311 (23.81)
	2	Contact	PM^a^, message	63 (4.82)
	3	Ship	Ship, shipping	68 (5.21)
	4	Scam	Scammer, scam, fake	83 (6.36)
	5	Product description	v1, v2, v3^b^, refill	53 (4.06)
	6	Purchasing method	eBay, website, .com, store	20 (1.53)
	7	Paying method	Gift card, PayPal, Bitcoin, BTC	40 (3.06)
	8	Stock	Still available, still selling, how many, still have, SOLD	15 (1.15)
	9	Flavor	Mango, mint, fruit, cucumber, crème, menthol, tobacco	27 (2.07)
	10	Other	Posts did not contain the words above	626 (47.93)
**JUUL** **subreddit (n=123)**				
	1	Buying/selling	This was annotated manually.	35 (28.46)
	2	Flavor	Mango, mint, fruit, cucumber, crème, menthol, tobacco	16 (13.01)
	3	Product description	Leak, refill, charged, help, real, fake, charger, battery	25 (20.33)
	4	Experience sharing	Buzz, cough, lung	9 (7.32)
	5	Other	Posts did not contain the words above	38 (30.90)

^a^PM: private message.

^b^v1, v2, and v3 represent the different versions of JUUL devices.

In the UnderageJuul subreddit, discussions were primarily focused on buying and selling JUULs. Topic 1 (311/1306, 23.81%) refers to users raising questions about the price. The replies usually contained specific prices in the form of $number. Topic 2 (63/1306, 4.82%) refers to posts in which authors would ask others to contact them through private messages if they did not want to directly answer questions in public replies. Topic 3 (68/1306, 5.21%) was about shipping services. Topic 4 (83/1306, 6.36%) includes posts in which some users commented under the threads with selling purposes that the post author is a scammer. Topic 5 (53/1306, 4.06%) refers to descriptions of the selling product, including the version of the product and whether it was refilled. Topic 6 (20/1306, 1.53%) and Topic 7 (40/1306, 3.06%) refer to the methods of purchasing and paying. Since some users self-claimed they were vendors from eBay or other websites, they accepted payment methods such as VISA gift cards and PayPal. Some users offered to buy JUULs from stores for underage users for a fee. Bitcoin was sometimes accepted as a form of payment. Topic 8 (15/1306, 1.15%) is related to users asking post authors about their stock. The post authors might update the threads with “SOLD” to indicate that they had sold out. Topic 9 (27/1306, 2.07%) represents the flavors discussed in the threads and comments; this topic is analyzed in detail in the next section.

In the JUUL subreddit, Topic 1 (35/123, 28.46%) represented the threads about buying and selling JUULs, which suggested the most popular topic discussed by these users did not change on this subreddit. Topic 2 (16/123, 13.01%) was about the flavors discussed by the overlapping users. The flavor topic is also analyzed individually in the next section. However, the focus of this topic was different from that on the UnderageJuul subreddit. Topic 3 (25/123, 20.33%) referred to the product descriptions. Unlike the product description topic (Topic 5) on the UnderageJuul subreddit, the discussions about this topic on the JUUL subreddit were about describing the device’s problems and seeking help. For instance, a post about this topic stated, “… All my official juul pods keep leaking out all over my juul. Idk what's going on.” Topic 4 (3/123, 2.44%) was about negative experience sharing. For example, one user said, “I used the juul to stop smoking but have noticed a chronic cough starting to begin.”

It can be seen that access to JUUL was a common topic for the discovered users in both subreddits. However, the content in the UnderageJuul subreddit included more details about JUUL transactions. The flavor topic and the product description topic appeared on both subreddits. The focal point in the UnderageJuul subreddit was users describing their buying/selling demands, while in the JUUL subreddit, users commonly shared their JUUL use experience.

We identified the 10 users who posted the most for each topic as the active users. In the UnderageJuul subreddit, there were 16 users who were active in multiple topics. There were 4 active users who were active in 3 topics at the same time. The most active user was in the top 10 among 6 topics. In the JUUL subreddit, there were 6 users active in multiple topics. The most active user posted about 4 topics at the same time. Notably, there were 7 users who were active in both subreddits, and the topics they promoted were related to JUUL buying and selling.

#### Flavor Analysis and Comparison

We counted the word frequency of the 7 flavors mentioned above and compared their popularity in the two subreddits. Among 382 threads and 1306 comments in the UnderageJuul subreddit, mango (n=50) was the most popular flavor, followed by cucumber (n=39) and mint (n=31). Fruit (n=9), tobacco (n=7), menthol (n=5), and crème (n=5) were not mentioned frequently. These flavors were mentioned when users described the selling products or the buying requests, as shown in the two examples given here:

Pods Available: I have 8 tobacco, 16 cucumber, 8 mint, 12 menthol, 8 mango, and 4 fruit. …

… Looking for 2 packs of mango pods let me know if you have any for sale.

Among the 123 threads in the JUUL subreddit, there were 16 threads that mentioned flavor. The most popular flavor was mango (n=8), followed by cucumber (n=7) and mint (n=7). These results were similar to those found in the UnderageJuul subreddit, which indicates that users’ flavor preferences did not change between subreddits. There was not much discussion of the crème (n=2), menthol (n=2), tobacco (n=2), and fruit (n=1) flavors. One example of a flavor topic post is as follows:

Just got my hands on a couple packs of cool cucumber. …, it's really good and up there with mango.

However, we found 3 reports of adverse symptoms among the 8 threads that mentioned the mango flavor in the JUUL subreddit. Two users reported mouth burning and one user reported a stomachache, as shown below:

…when ever I hit a mango pods my stomach almost immediately starts hurting.

### Posting Activeness Observation

We observed the posting activeness of all labeled users in the year following the shutdown of the UnderageJuul subreddit (January 8, 2018, to January 8, 2019). Table 8 shows the number of labeled users who had posted during this time period, and Table 9 shows the number of posts they made. For both tables, values were summed across consecutive 2-month periods.

**Table 8:** The number of users who posted messages in the year after the UnderageJuul subreddit was removed.

Time period	Users who wanted to buy	Users who wanted to sell	Self-reported underage users
1/8/2018-3/7/2018	11	12	4
3/8/2018-5/7/2018	5	8	0
5/8/2018-7/7/2018	2	1	0
7/8/2018-9/7/2018	1	2	1
9/9/2018-11/7/2018	0	6	0
11/8/2018-1/9/2019	2	3	1

**Table 9:** The number of posts by the labeled users in the year after the UnderageJuul subreddit was removed.

Time period	Posts by users who wanted to buy	Posts by users who wanted to sell	Posts by self-reported underage users
1/8/2018-3/7/2018	34	48	17
3/8/2018-5/7/2018	12	24	0
5/8/2018-7/7/2018	6	3	0
7/8/2018-9/7/2018	1	3	1
9/9/2018-11/7/2018	0	23	0
11/8/2018-1/9/2019	4	28	2

In the first 4 months after the UnderageJuul subreddit was shut down, the discovered users were still active in the JUUL subreddit. They posted 10 threads with the purpose of buying or selling. In the next 2 months, these users were less active and did not post threads with buying or selling willingness. One possible reason was that the administrators of the JUUL subreddit started to prohibit posts about trading JUULs. In the second half of the year, the discussions changed to flavor and device-related topics. There were 2 users who expressed the intention of quitting JUULs because of nicotine addiction and the high price.

## Discussion

### Principal Findings

This paper used a cross-subreddit method to analyze underage JUUL use. Based on our previous study [17], this paper further analyzed the content related to underage JUUL buying and selling on Reddit, and summarized its characteristics and patterns. Additionally, this paper analyzed the posting activeness of the discovered users in the JUUL subreddit after long-term observation.

In summary, we found 214 threads related to JUUL sale and 168 threads related to JUUL purchase on the UnderageJuul subreddit, which accounted for 53.5% (382/714) of the UnderageJuul subreddit’s threads. In addition, we found that these threads were posted with certain regular word-level patterns. The most common key phrases used in selling-related threads were “WTS,” “want to sell,” “for sale,” and “selling” (154/214, 72.0%). The most common key phrases used in buying-related threads were “look for/get JUUL/pods” (58/168, 34.5%) and “WTB” (53/168, 31.5%). The FDA has already announced policies about preventing youth use of and access to JUUL e-cigarettes, and reducing the marketing and promotion of tobacco products toward minors [29,30]. Though the UnderageJuul subreddit has been removed, there might be other similar communities that support underage JUUL trading. The posting patterns could help discover this type of content among other communities on Reddit and different social media platforms such as Twitter and Instagram. Once such content is discovered, a link to the official educational page about the tobacco products’ adverse health effects could be automatically added below such posts or sent to the content publisher via private message. This could have implications for interventions to reduce youth ENDS use.

We have demonstrated that discussions about obtaining and using JUUL e-cigarettes occur in multiple venues, and not just the subreddit dedicated to underage JUUL use (UnderageJuul). Among the 844 Redditors of the UnderageJuul subreddit, 23.82% (201/844) of users were annotated with buying intention, and 21.10% (178/844) of users were annotated with selling intention. We found that 24.38% (49/201) of users who wanted to buy JUULs and 46.63% (83/178) of users who wanted to sell JUULs had posting activeness on the JUUL subreddit. The results are consistent with our earlier research [17] regarding the approaches of illegally obtaining JUUL products. The buying/selling topics were common concerns for users on both subreddits during the same time span. This indicates the people who want to access to JUULs may not express their desires on only one platform and may seek multiple resources. When regulatory organizations take action to reduce youth’s access to JUUL and similar products, they might need strategies on multiple social media platforms simultaneously. When selling ENDS products via online shopping sites and other vaping-related websites, necessary warning statements should be clearly presented alongside, including the health risks and nicotine strength [31].

In our cross-subreddit analysis, we developed a procedure to evaluate the effect that the discovered users had based on their posting activeness and user-generated content. The most active self-reported underage user we discovered had published content that at least 128 Redditors read about, so there was a social network effect that could impact youth use of ENDS products like JUUL. Evaluation of JUUL-related content posted by those active users on social media platforms, especially the underage ones, is highly relevant to educators and those involved in tobacco regulation. In addition, it is not clear from these analyses whether these active users are paid by vendors or companies for advertising tobacco products to minors, and this is an important analysis that should be conducted to understand the motivation of those who post about these products on social media.

Our findings suggest that price was what the UnderageJuul users most cared about, along with the delivery services. The mango, mint, and cucumber flavors were the favorite flavors among these users. In January 2018, the UnderageJuul subreddit was banned. We found there was a self-reported underage user that posted a message on the JUUL subreddit asking for an alternative to the mango flavor, such as mint. In addition, we discovered negative health symptoms were reported on the JUUL subreddit after the use of the mango flavor. These popular flavors are still available in many countries and young people continue to discuss ways to acquire ENDS products like JUUL on Twitter, Reddit, etc. Despite regulatory and company efforts to reduce access to flavored ENDS products [32], considerable research is still needed on the role of flavors regarding the addiction potential of ENDS products [13,18,19,33]. In particular, since social media can provide timely and rapid information on what is being discussed about a particular ENDS product, continued analysis of social media is an essential early warning surveillance method for identifying addiction and the health risks of JUUL and other products (eg, the NJOY Ace product that has steadily increased in sales).

In our posting activeness observation, we found that in the year after the UnderageJuul subreddit was removed, 40 users (including 4 self-reported underage users) continued to post 186 threads in the JUUL subreddit. There were 10 threads with buying/selling willingness during the first 2 months. This result indicates that shortly after the UnderageJuul subreddit was closed, users transferred to other subreddits to have discussions, and continued to publish messages about buying/selling JUUL pods through social media platforms.

In conclusion, this study highlights the need for continued research on how social media can become a more fundamental component of the tobacco and nicotine surveillance system. In particular, rapid and extensive analyses of social media can serve as an early warning system of the rapid rise in the use of new products and health problems that are occurring as a result of using those products.

### Limitations and Future Work

Since the data we used for analysis was user-generated public information (ie, post, publication date, and username), there were many limitations for us regarding determining the authenticity and legitimacy of users’ claims like self-reported age, their stock of JUUL devices, and the completion of trading. As the UnderageJuul subreddit was built specifically for discussing underage JUUL use, we thought the age distribution analysis was valuable and necessary. In addition, we noticed the scam topic (ie, Topic 4 on the UnderageJuul subreddit on Table 7) during the content analysis, which indicates some Redditors who claimed to have JUULs to sell might post such content to scam other users, rather than to trade JUULs. This might cause discrepancies for regulators when trying to identify actual buyers and sellers.

In addition, the conclusions and insights of our study were based on content analysis of the two specific subreddits during the specific time span. Our findings might not be generalizable to other subreddits or other time periods, considering the different barrier policies being carried out. However, future studies could be extended to other subreddits that are about JUULs and other social media platforms (eg, Twitter) with the same methodology and mechanism.

### Conclusion

This is the first study to investigate the patterns of buying and selling among underage JUUL users by utilizing information from multiple connected online forums. We hope these findings can be conducive to timely guidance and education of underage JUUL users, thus protecting the health of young people.

## Abbreviations

API: application programming interface
ENDS: Electronic Nicotine Delivery Systems
FDA: Food and Drug Administration
